# You are exactly my type! The traits of a good doctor: a factor analysis study on public's perspectives

**DOI:** 10.1186/s12913-022-08273-y

**Published:** 2022-07-08

**Authors:** Julia S. Grundnig, Verena Steiner-Hofbauer, Viktoria Drexler, Anita Holzinger

**Affiliations:** Teaching Center, Medical University of Vienna, Spitalgasse 23, BT 87, Vienna, Austria

**Keywords:** Medical professionalism, Doctor–patient relationship, Public views, Attributes, Qualities, Physician behaviour

## Abstract

**Background:**

A multiplicity of qualities and behaviours are considered essential in a good doctor and are identified in various medical profession frameworks. However, there is no consensus as to their meaning or even agreement on fundamental qualities. The authors wanted to examine the importance placed by the Austrian public on the professional and personal traits of ideal physicians. Competencies were used to create different types of ‘good doctor’ and then examined to discover how these can be integrated into existing medical professionalism frameworks.

**Methods:**

A 69-item Likert scale-based questionnaire was developed and administered via telephone interview to 1,000 subjects. Computer-assisted telephone interviews (CATI) were carried out by the Austrian GALLUP-Institute. An explorative factor analysis with promax rotation was undertaken to summarise the interrelationships among variables.

**Results:**

Factor analysis identified six interpretable factors which we define as six different types of doctors: the dutiful doctor, the online health-celebrity, the medical expert, the service physician, the medical altruist, and the ethical agent. The items perceived as most important were ‘takes time’, ‘listens’, and ‘makes correct diagnoses’. Outcome measures of internal consistency and reliability estimates (Cronbach´s alpha, 0.69–0.86) for each element.

**Conclusions:**

The six types of physicians may be a step toward recognizing the professional behaviour of all physicians, their actions as healers, and their commitment to moral concepts, values, and needs of their patients, and society. According to our results, the public has expectations of good doctors that go beyond the scope within the medical professionalism frameworks. Therefore, these guidelines should be adapted in light of the changing expectations and needs of the general population.

## Background

The ultimate goals of medicine can only be achieved when good doctors practise good medicine and when all those participating in medical care are satisfied. Good doctors must be grounded in their profession and should express attributes that match social expectations [1]. Medical professionalism (MP) thus forms the basis of the relationship between society and its doctors by defining the set of values and behaviours expected. The qualities of MP were conceived by doctors, for doctors [2]. However, does the public value these qualities in the same way? What qualities make a doctor a good doctor to the people she/he treats or to the public she/he serves?

As the medical profession considers its role in society, defining what is meant by medical professionalism has become increasingly important. However, the concept of ‘medical professionalism’ is constantly evolving [3]. The concept has been changing since the mid-1960s, primarily due to the inherent conflict between the altruism expected of MP, the self-interests of doctors and increasingly, the economic orientation and bureaucratisation of the healthcare system [4]. While doctors’ perspectives may have remained fairly consistent, the healthcare expectations of an increasingly well-informed consumer society have changed significantly [5]. Medicine has become a marketplace for patients, doctors, the pharmaceutical industry, insurance companies and the health departments of governments, and the scope is constantly expanding [6]. Consequently, people increasingly have their concepts of what constitutes a good doctor-patient relationship [7]. Clarification regarding expectations of medical health care is a fundamental first step.

Various definitions of MP have been provided by major medical organizations [8]. The General Medical Council (GMC), in its publication ‘Good Medical Practice’, describes the four responsibilities of a physician as: ‘knowledge, skills, and performance’, ‘safety and quality’, ‘communication, partnership, and teamwork’ and ‘maintaining trust’ [9]. The American Board of Internal Medicine (ABIM), the American College of Physicians (ACP) and the European Federation of Internal Medicine (EFIM) have together developed a ‘Physician Charter’, which contains three fundamental principles and ten professional responsibilities. The charter ‘supports physicians’ efforts to ensure that healthcare systems and the physicians working within them remain committed to both to patient welfare and to the basic tenets of social justice’ [10]. The ABIM defines MP in terms of altruism, accountability, excellence, duty, integrity, and respect [10]. Another framework for medical competencies has been put forward by CanMEDS and The Royal College of Physicians and Surgeons of Canada describes seven distinct roles for a good doctor: medical expert, communicator, collaborator, leader, health advocate, scholar, and professional [11, 12].

Medical professionalism should be deeply embedded in the self-concept of every doctor [13]. Physicians possess a wide range of skills and attributes and are expected to act as professional role models. The internationally recognized guidelines for MP [10, 14, 15] can be used as objective criteria for what is expected of a good doctor. Whether these are formal statutes or voluntary commitments, all attempt to arrive at an objective and unbiased standard of medical professionalism. Governments, universities, health insurance and politicians, as well as those involved in the medical care system, are interested in the attributes that together constitute a good doctor [16]. The definition of a good doctor will depend on who is being asked [17]. There has been increasing emphasis on the attributes that patients [2, 18], physicians [19, 20], and medical students [8, 21, 22] value in a good doctor. These may differ from what the broader public considers important. In our opinion, the final arbiter should be the members of the public as they are on the receiving end of medical care.

To date, several studies have reported perceptions of MP as seen by different stakeholders, but only a few studies have explored the public’s perspective on the constructs ‘medical professionalism’ and the ‘good doctor’ [3, 20, 23–25]. A paper by Cruess and Cruess provides a helpful overview of the expectations and obligations of the various parties [17]. Members of the public are shown to value interpersonal relations and technical skills in their physician; they appreciate humanity, expertise, being listened to, being provided with information about their disease and treatment prospects, being given adequate consultation time and being involved in decisions relating to their care [26, 27]. However, if one looks further than the medical frameworks or guidelines, it is difficult to shake off the impression that the traditional relationship between doctors and society is undergoing a profound change.

This study aimed to determine the qualities that a cross-section of the Austrian general public considers important in their doctors. Therefore, we will provide a new perspective on the concept of a good doctor by examining the various professional and personal factors that together constitute good medical practice in the 21^st^ century. We also intend to outline how these different factors can be integrated into the three existing MP frameworks: CanMEDS, Physician-Charter, and GMC. The study will produce a ranked and rated list of human qualities and professional behaviours that the public considers the most important. These competencies are then combined to suggest different types of a good doctor. We used this approach to stay as close to the existing frameworks as possible but to extend them to identify types of doctors that match the wishes and needs of the public. The types of a doctor presented describe typical physician roles as understood by the public.

## Methods

### Data collection and sample

The research presented here is part of a mixed-methods survey conducted by the Medical University of Vienna. A representative anonymous telephone study of 1,000 participants was carried out in Austria in February and March 2020. The criteria for representativeness were: sufficiently high number of cases; comparatively small ranges of variation of +/- 1.4 to +/- 3.2 for a representative sample of n = 1,000 interviews; simple random sampling; and each person in the population has the same chance of becoming part of the sampling. The achieved sample represents the Austrian population on as many socio-demographic dimensions as possible. To ensure representativeness, the sample was quota-ranked according to the variables of gender, age, federal state, educational background, and city size. The study has been conducted according to Guidelines of the Helsinki Declaration of Good Clinical Research Practice

Randomised phone interviews were conducted through an experienced private marketing and research institute (Austrian GALLUP-Institute), using computer-assisted telephone interviewing (CATI). Respondents were selected through the randomised last digit method, which generates random numbers. The interviews were conducted in German and lasted approximately 14 minutes.

The sample is considered representative of the demographics, gender, age, education, and federal state. The age of the respondents ranged from 18 to 75 years (M = 46.37; SD = 15.8). Of the 1,000 respondents, 51.5% were women and 48.5% were men (Table 1).

**Table 1:** Sample characteristics (*n* = 1,000); 2020 Good Doctor Survey

Characteristic	Participants (N, %)
Sex	
Female	515 (51.5%)
Male	485 (48.5%)
Age Group (years)	
18-30	215 (21.5%)
31-40	168 (16.8%)
41-50	188 (18.8%)
51-60	215 (21.5%)
61-75	214 (21.4%)
Country of Birth	
Austria	963 (96.3%)
Other	37 (3.7%)

### Questionnaire

The questionnaire was developed to measure the Austrian public’s expectations of good doctors. An initial pool of 71 items was collected from a literature review [28], personal theory, and educational practice. Participants were asked to rate the importance of each item on a five-point Likert scale (1 = very important to 5 = not important at all). To test the comprehensibility and adequacy of the items with regard to different population groups, a pre-test was conducted with 20 persons. The pre-test showed that some items were formulated imprecisely and thus could not be understood. Therefore, a revision of the questionnaire was necessary and the number of items was reduced to 69.

### Statistical analysis

Descriptive analyses were used to compute median scores, standard deviations, and item variances. An exploratory factor analysis with promax rotation and Kaiser normalization was conducted to examine the structure underlying the 69 items. Assumptions regarding the normality of the distribution were met, as assessed by the Shapiro-Wilk-Test. The Kaiser criterion was used to drop the least important factors with eigenvalues > 1.0. Internal consistency and reliability were determined by employing Cronbach’s alpha. Bartlett’s test of sphericity, which tests the overall significance of all the correlations within the correlation matrix, was significant (χ2 (2346) = 16019.31, p<0.001), indicating that it was appropriate to use the factor analytic model on this set of data. The Kaiser-Meyer-Olkin measure verified the sampling adequacy for the analysis, KMO = .89, where all KMO values for individual items were >.72, which is well above the acceptable limit of .5 [21]. For missing data, list-wise exclusion was chosen because no variable showed more than 7% missing values, and 72% of all cases (*n* = 719) showed no missing values. Fifteen factors had eigenvalues over the Kaiser criterion of 1 and explained 56% of the variance. The scree plot was ambiguous and showed inflexions justifying four or six factors. Discussion among all authors and their consensus determined the final number of factors. All six scales had alpha reliability ranging from 0.69 to 0.86. Data was analysed using SPSS 28.0 for Windows (IBM, SPSS, Armonk, New York).

## Results

Table 2 summarises the descriptive data of the items comprising the concept of a good doctor. The list starts with items rated as the most meaningful and catalogues them in order of perceived importance by ascending order of median. Of the items, 80% (55/69) were rated important and had an average score of two or lower. The three items perceived as most important were ‘takes time’, ‘listens’, and ‘makes correct diagnoses’. The three least meaningful items were ‘considers she/he can only examine in private practice’, ‘is good-looking’, and ‘is well-known from TV or the media’. These ratings were consistent across gender and age. There were no significant gender differences. Table 2 shows the median and standard deviations; all elements are negatively skewed.

**Table 2:** Median scores for 69 items ‘good doctor survey’, listed by degree of perceived importance

	Components	Median	Std. Dev.	Missing
1.	Takes time	1	0.424	3
2.	Listens	1	0.413	0
3.	Makes correct diagnoses	1	0.410	2
4.	Conveys the feeling of being in safe hands	1	0.477	1
5.	Strictly adheres to medical confidentiality	1	0.515	3
6.	Gives detailed information on diagnosis and therapy	1	0.531	1
7.	Tells the truth, even if the diagnosis is unpleasant	1	0.564	2
8.	Has a heart for people	1	0.540	1
9.	Can admit it if she/he makes a mistake	1	0.567	8
10.	Can empathise with patients	1	0.575	2
11.	Has broad medical knowledge	1	0.590	2
12.	Knows what he/she can and cannot do	1	0.583	4
13.	Takes patients’ explanations of disease origin seriously and considers them in prescribing therapy	1	0.601	2
14.	Is patient	1	0.600	2
15.	Collects a comprehensive medical history	1	0.620	3
16.	Maintains detailed patient documentation	1	0.646	2
17.	Involves patients in decision-making	1	0.664	3
18.	Provides information about preventive measures	1	0.654	1
19.	Is not influenced by pharmaceutical companies	1	0.741	13
20.	Is resilient	1	0.673	0
21.	Radiates optimism	1	0.675	0
22.	Considers surgery as a last resort after all other treatment options have been exhausted	1	0.705	10
23.	Assigns appointments promptly	1	0.731	2
24.	Treats all patients equally, regardless of their social or cultural background	1	1.165	4
25.	His/her treatment is based on the latest scientific findings	1	0.778	12
26.	First examines the patient thoroughly physically (e.g. with a stethoscope, palpation) before she/he carries out instrument-based examinations (e.g. X-ray, MRI)	1	0.783	7
27.	Participates regularly in advanced medical training	1	0.906	5
28.	Remembers the patient’s medical history well	1	0.833	3
29.	Takes account of the patient’s family and personal concerns	1	0.848	9
30.	Does not make patients wait long for their appointment	1	0.851	2
31.	Is assertive and resolute	2	0.777	5
32.	Leads a publicly accessible non-private practice	1	0.982	8
33.	Separates medical profession from his or her personal life	1	0.963	19
34.	Offers house calls	1	0.988	2
35.	Is altruistic and puts financial interests in the background	2	0.943	5
36.	Adjusts payment in his/her private practice to fit patients’ financial situation	2	1.111	32
37.	Has many years of professional experience	2	0.983	5
38.	Has a good sense of humour	2	0.991	2
39.	Offers opening hours on weekends and in the evening	2	1.095	3
40.	Offers alternative medicine (e.g. homeopathy, osteopathy)	2	1.166	10
41.	Sacrifices himself/herself for the profession	2	1.110	5
42.	Sets a good example (e.g. does not smoke, drinks little alcohol, does sport regularly)	2	1.198	13
43.	Does not waste many words but decides quickly	2	1.255	5
44.	Is specialized in a medical discipline	2	1.155	14
45.	Favours mandatory vaccination	2	1.272	13
46.	Favours digital medical files	2	1.197	38
47.	Is unwilling to prescribe a drug contrary to his/her medical expertise, even if the patient asks him/her to do so	2	1.244	13
48.	Advocates lower health insurance contributions if someone verifiably lives healthily	2	1.276	31
49.	Strictly follows conventional medicine	2	1.232	29
50.	Is unwilling to conduct examinations requested by patients if there is no medical justification for doing so	2	1.247	26
51.	Is against the artificial prolongation of life	3	1.304	62
52.	Offers an in-house pharmacy	3	1.451	5
53.	Has an internet homepage	3	1.335	4
54.	Communicates with patients via e-mail	3	1.319	11
55.	Has good online reviews	3	1.428	17
56.	Has recently completed his/her medical education and is up to date	3	1.227	16
57.	Advocates euthanasia, i.e. is willing to assist medically with suicide at the patient’s request (for example: Switzerland)	3	1.380	55
58.	Advocates that everyone is automatically an organ donor without being asked	3	1.507	49
59.	Prescribes painkillers and sedatives quickly and easily	3	1.229	6
60.	Prefers to work in a group practice or a healthcare centre rather than in an individual practice	3	1.291	32
61.	Has made medicine a career (e.g. is a professor or medical head of a department)	3	1.356	6
62.	Hands out free samples of medication	3	1.379	11
63.	Accepts patients by referral only	3	1.325	23
64.	Issues prescriptions online	4	1.344	16
65.	Gives patients a sick note quickly and easily at their request	3	1.271	15
66.	Offers online therapy	4	1.272	31
67.	Considers he/she can only examine well in private practice	4	1.337	24
68.	Is good-looking	4	1.267	8
69.	Is well-known from TV or the media	5	1.242	3

Median Scores computed from Likert scale given to 69 items of the good doctor questionnaire; listed by degree of perceived importance (*N* = 719); Scale: 1: Very Important; 2: Important; 3: Somewhat important; 4: Not important; 5: Not important at all

A principal component analysis of item intercorrelations was carried out for the 719 complete sets of data to generate factors. The analysis confirmed six principal components, which accounted for 56% of the variance. Only four items were missing from the set. Items were assigned based on loadings of 0.30 or greater (see Table 3). Despite high levels of overall commonality, six factors emerged, each with its own distinct facet of the good doctor. The names given to the factors reflect a holistic and substantive interpretation process. Factor 1 consists of 17 items (α = .81) and represents ‘the dutiful doctor’; Factor 2 consists of 13 items (α = .86) and represents ‘the online health-celebrity’; Factor 3 consists of 8 items (α = .80) and represents ‘the medical expert’; Factor 4 consists of 8 items (α = .69) and represents ‘the service physician’; Factor 5 consists of 13 items (α = .78) and represents ‘the medical altruist’; and Factor 6 consists of 6 items (α = .73) and represents ‘the ethical agent’.

**Table 3:** Summary of factor analysis results for the ‘good doctor’ questionnaire (*N* = 719)

Components	Factor 1: Dutiful Doctor	Factor 2: Online Health Celebrity	Factor 3: Medical Expert	Factor 4: Service Physician	Factor 5: Medical Altruist	Factor 6: Ethical Agent
Maintains detailed patient documentation	**0.397**	0.077	0.043	-0.042	0.225	-0.058
Strictly adheres the medical confidentiality	**0.338**	-0.053	0.046	-0.027	0.036	-0.141
Is not influenced by pharmaceutical companies	**0.451**	0.006	0.052	0.023	-0.012	0.025
Takes time	**0.584**	0.063	-0.091	0.090	-0.067	0.024
Listens	**0.670**	0.055	-0.107	-0.010	-0.035	0.071
Has broad medical knowledge	**0.417**	-0.106	0.279	0.024	0.095	0.143
Makes correct diagnoses	**0.504**	-0.014	0.072	-0.251	0.100	-0.026
Collects a comprehensive medical history	**0.437**	0.091	-0.005	0.000	0.218	-0.019
Provides information about preventive measures	**0.359**	-0.051	0.163	0.015	0.210	0.047
Gives detailed information on diagnosis and therapy	**0.558**	-0.076	0.090	0.004	-0.005	0.014
First examines the patient thoroughly physically (e.g., with a stethoscope, palpation) before she/he carries out instrument-based examinations (e.g., X-ray, MRI)	**0.393**	-0.001	0.019	0.239	0.114	0.144
Considers surgery as a last resort after all other treatment options have been exhausted	**0.392**	0.052	-0.013	0.090	0.147	-0.019
Involves patients in decision-making	**0.441**	0.151	0.069	0.096	0.075	-0.125
Can empathize with patients	**0.501**	-0.008	-0.036	0.093	0.086	-0.093
Tells the truth, even if the diagnosis is unpleasant	**0.418**	0.005	0.117	-0.073	-0.003	-0.066
Takes patients' explanations of disease origin seriously and considers them into therapy	**0.393**	0.060	0.179	0.063	0.014	-0.163
Can admit, if he/she makes a mistake	**0.428**	0.092	0.039	-0.183	0.051	-0.206
Sets a good example (e.g., does not smoke, drinks little alcohol, does sport regularly)	0.090	**0.303**	0.164	-0.005	0.185	-0.058
Is good-looking	-0.207	**0.432**	-0.157	0.175	0.227	0.123
Is well-known from TV or the media	-0.247	**0.402**	-0.024	0.237	0.132	0.264
Has good online reviews	-0.002	**0.354**	0.328	0.072	0.112	-0.092
Offers online therapy	0.103	**0.848**	0.026	-0.039	-0.133	0.091
Issues prescriptions online	0.140	**0.898**	0.019	-0.146	-0.194	0.011
Communicates with patients via e-mail	0.200	**0.828**	-0.218	-0.110	-0.049	0.183
Gives patients a sick note quickly and easily at their request	-0.085	**0.507**	-0.239	0.334	-0.043	-0.076
Prescribes painkillers and sedatives quickly and easily	-0.156	**0.564**	0.076	-0.014	0.027	-0.025
Accepts patients by referral only	-0.082	**0.662**	0.038	-0.126	0.003	0.137
Has an internet homepage	0.157	**0.715**	0.081	-0.163	-0.073	0.115
Considers he/she can only examine well in private practice	-0.164	**0.457**	0.034	0.142	0.029	0.329
Prefers to work in a group practice or a health care center rather than in an individual practice	-0.037	**0.357**	0.040	0.324	-0.097	0.332
Treats all patients equally, regardless of their social or cultural background	0.202	-0.134	**0.748**	0.001	-0.207	0.326
Favors digital medical files	0.063	0.215	**0.436**	-0.090	-0.005	0.111
Favors mandatory vaccination	-0.114	0.124	**0.507**	-0.253	0.143	0.246
His/Her treatment is based on the latest scientific findings	0.268	0.055	**0.583**	-0.058	-0.087	0.082
Is specialized in a medical discipline	-0.098	-0.063	**0.520**	0.270	0.055	0.001
Has made a career (e.g., is a professor or a medical head of a department)	-0.094	0.382	**0.404**	0.155	-0.010	-0.173
Does not waste many words but decides quickly	-0.098	-0.034	**0.696**	0.105	0.086	-0.012
Participates regularly in advanced medical training	0.176	-0.157	**0.760**	0.022	-0.124	0.214
Advocates lower health insurance contributions if someone verifiably lives healthy	0.050	0.023	0.279	**0.387**	-0.018	-0.008
Offers alternative medicine (e.g., homeopathy, osteopathy)	0.308	0.131	-0.092	**0.481**	-0.126	0.289
Offers opening hours on weekends and in the evening	0.202	0.233	0.301	**0.303**	-0.244	-0.212
Leads a publicly accessible non-private practice	0.060	-0.035	-0.164	**0.437**	0.180	-0.137
Offers an in-house pharmacy	-0.110	-0.163	0.166	**0.626**	0.182	0.023
Hands out free samples of medication	-0.153	0.028	0.177	**0.560**	0.077	0.103
Offers house calls	0.064	-0.241	-0.075	**0.750**	0.043	0.108
Has many years of professional experience	-0.049	0.039	0.102	**0.423**	0.080	-0.198
Follows strictly conventional medicine	-0.179	0.193	0.327	-0.191	**0.336**	-0.320
Has a heart for people	0.402	-0.112	-0.038	0.065	**0.415**	0.206
Sacrifices himself/herself for the profession	-0.051	0.000	0.254	0.181	**0.374**	-0.149
Knows what he/she can and cannot do	0.232	-0.037	0.056	-0.218	**0.464**	-0.076
Does not make patients wait long for their appointment	0.075	0.019	-0.070	0.263	**0.344**	-0.172
Is altruistic and puts financial interests in the background	0.109	-0.050	-0.015	0.162	**0.462**	-0.060
Is assertive and resolute	0.026	-0.221	0.433	0.039	**0.489**	0.109
Conveys the feeling of being in safe hands	0.380	-0.036	-0.028	-0.142	**0.458**	0.103
Is resilient	0.157	-0.166	0.026	0.037	**0.633**	0.143
Has a good sense of humor	-0.002	0.024	-0.071	0.111	**0.640**	0.312
Is patient	0.316	0.005	-0.171	0.002	**0.529**	0.008
Radiates optimism	0.294	-0.065	-0.071	0.167	**0.514**	0.137
Takes account of the patient's family and personal concerns	0.211	0.047	-0.068	0.177	**0.356**	-0.103
Is against the artificial prolongation of life	0.098	-0.026	0.147	0.254	-0.004	**0.535**
Advocates euthanasia, i.e., is willing to assist to medically suicide at the patient's request (example: Switzerland)	-0.015	0.139	0.141	0.054	0.041	**0.557**
Advocates that everyone is automatically an organ donor without being asked	-0.124	0.154	0.094	-0.065	0.086	**0.633**
Is unwilling to prescribe a drug contrary to his/her medical expertise, even if the patient asks her/him to do so	0.021	0.317	0.055	-0.289	0.161	**0.574**
Is unwilling to conduct examinations requested by patients if there is no medical justification for doing so	-0.069	0.367	0.016	-0.172	0.231	**0.486**
Has recently completed his/her medical education and is up to date	-0.127	0.153	0.319	0.254	0.000	**0.357**
Separates the medical profession from his or her personal life	0.046	0.016	0.277	0.050	0.257	-0.056
Adjusts payment in his/her private practice to fit patients financial situation	0.187	0.254	-0.047	0.104	0.128	-0.241
Assigns appointments promptly	0.270	0.195	-0.045	0.068	0.118	-0.283
Remembers the patient's medical history well	0.232	0.129	-0.033	0.242	0.136	-0.258
Eigenvalues	9.99	7.07	3.34	2.28	1.56	1.45
% of variance	14.49	10.24	4.83	3.31	3.17	2,26
α	0.81	0.86	0.80	0.69	0.78	0.73

Factor loadings of 6 factors loaded by 69 items of ‘good doctor survey’ Extraction method: principal component analysis, rotation method: promax with Kaiser Normalization, rotation converged in 14 iterations. Note: Factor loading appears in bold if it is the highest loading of all six factors and at the same time is above 0.3

### Factor 1: The dutiful doctor

Being a dutiful doctor requires taking time, listening, giving detailed information about both diagnosis and therapy, and providing information about preventive measures. This type of doctor examines the patient thoroughly before carrying out instrument-based examinations, makes correct diagnoses, and has broad medical knowledge. He collects a comprehensive medical history, maintains detailed patient documentation, and can admit if he has made a mistake. The dutiful physician empathizes with patients, involves them in decision-making, takes their explanations of their disease’s origins seriously, and considers them in prescribing therapy. Furthermore, this type of doctor is not influenced by pharmaceutical companies, adheres strictly to medical confidentiality, tells the truth, even if the diagnosis is unpleasant, and considers surgery to be a last resort after all other treatment options have been exhausted.

### Factor 2: The online health celebrity

The celebrity doctor offers online therapy, issues prescriptions online, and communicates via e-mail. This physician is well known on TV or media, has a homepage, has good online reviews, is good-looking, and sets a good example. He considers working in private practice, but prefers to work in a group practice or in a healthcare centre where patients are accepted only by referral. This type of doctor gives sick notes on request and prescribes painkillers and sedatives quickly and easily.

### Factor 3: The medical expert

The medical expert treats all patients equally, regardless of their social or cultural background, participates regularly in advanced medical training, has specialized in a medical discipline, and has made a career of medicine. The treatment these doctors provide is always based on the latest scientific findings; they favour digital medical files and mandatory vaccination, and do not waste many words but make decisions quickly.

### Factor 4: The service physician

Service physicians provide house calls, an in-house pharmacy, and opening hours on weekends and in the evening. They have many years of professional experience and lead a publicly accessible, non-private practice. They hand out free medication samples and offer alternative medicine. These doctors advocate lower health insurance contributions if someone verifiably lives healthily.

### Factor 5: The medical altruist

Being an altruistic physician means being patient, resilient, altruistic, assertive, and resolute. This type of doctor has a heart for people, sacrifices herself/ himself for the profession, conveys the feeling of being in safe hands, has a good sense of humour, and radiates optimism. These practitioners strictly follow conventional medicine, know what they can and cannot do, take patients’ family and personal concerns into account, and do not make patients wait for their appointments.

### Factor 6: The ethical agent

Ethical physicians are characterised by the belief that everyone is automatically an organ donor without being asked. They advocate euthanasia and are against the artificial prolongation of life. They have completed their medical education recently and are therefore up-to-date. In addition, they are unwilling to prescribe drugs that go against their medical beliefs, even if the patient asks them to do so, and they will not conduct examinations as requested by a patient if there is no medical justification for doing so.

## Discussion

This study aimed to outline the different types of a good doctor and to shed light on how the adult population of Austria responds to the question ‘What makes a good doctor?’. Factor analysis showed six related factors. Hence, this study offers valuable insights into the Austrian public’s perceptions of the different physicians’ qualities. To the best of our knowledge, this is the first study to consider the topic from this perspective. The general population values communication and patient-centred care, as well as integrity and clinical ethics. When all types of doctors are considered together, a picture of the good doctor emerges: according to this, the general population expects their physician to be dutiful, altruistic, and motivated by ethical principles. At the same time, good doctors should be accessible online, have medical expertise, and offer a range of medical services.

The study revealed two new findings: First, our six doctor types may be a step towards recognizing the professional behaviours of all physicians, their actions as healers, and their commitment to moral concepts, to their patients’ values and needs, and to society [17, 23]. Second, the public does not equate the medical profession with social standing, wealth accumulation or physical characteristics. All ratings were consistent across gender, age and social class.

In the context of medical professionalism, which is increasingly seen as a social contract [8], the public assesses doctors as ‘good’ due to their moral behaviour, high values, and positive attitudes. The general population expects doctors to be confident, reliable, dependable, composed, accountable, and dedicated in all situations. Personal appearance, physical characteristics, social status, and practice habits play little or no role in determining whether a doctor is classified as ‘good’.

In line with previous studies, the public values good interpersonal relationships, professional skills, humanity, and competence in a doctor. They want to be listened to, to be provided with full information about their illness and treatment options, to be given sufficient time during consultations, and to be involved in decisions relating to their treatment [26, 27]. Literature focusing on the public’s perspectives [3, 20, 23–25] reveals the high importance of interpersonal qualities, such as communication skills, empathy, compassion, and a caring attitude. In addition, it emphasises doctors’ knowledge and performative skills. Recent research points to qualities that include both cognitive and non-cognitive skills, such as integrity, empathy, and social skills [29].

The doctor types provide descriptions of particular characteristics or qualities. These factors can be understood as six essential competencies of all physicians. Items within the factors are not exhaustive, and there may be other important characteristics that are not included. The types are not mutually exclusive, but are a manifestation of the range of expectations people have of a good doctor. Becoming a good doctor is consistent with each factor in this analysis. Although we have presented these six as pure types, the edges are often diffuse: ideally, physicians should combine all types or move among them. Being a medical professional requires conscious and continuous maintenance of all these facets of medical practise.

Both overlap and differences are evident when comparing our six types with CanMEDS, GMC and the Physician Charter. Most of the contents of those frameworks [9–11] could be categorized under the types identified in this study. Our factors provide a helpful pattern for conceptualising the various facets of medical professionalism. Almost all the items in each type are consistent with the components of other frameworks. However, statements about having a sense of humour, offering alternative medicine, having a homepage and online reviews, and being attractive or well-known are not present in all MP guidelines. The types ‘dutiful doctor’, ‘medical expert’, ‘ethical agent’, and ‘medical altruist’ correspond most closely with other concepts of MP. However, none of those approaches mention taking time and empathizing with patients. These two aspects are important to the general public and their inclusion should therefore be considered. The types ‘service physician’ and ‘online health-celebrity’ are less well-matched against the three other frameworks.

The contents of all frameworks overlap, but it is easiest to identify our types in the roles described in CanMEDS [12]. These competencies are likely to be associated with a particular aspect of medical practice. Significant links between our results and the CanMEDS roles are communicator, medical expert, health advocate, and professional. Our questionnaire had few items relating to collaboration with colleagues, nursing staff or other health care professionals, to management or leadership skills, to scholarly abilities, to evaluating evidence or to teaching others. Therefore, the CanMEDS roles of collaborator, leader, and scholar were not represented in our data.

There are links between our typology and the concepts of Good Medical Practice [9]. Our physician types cover the four domains: knowledge, skills, and performance; safety and quality; communication, partnership and teamwork; and maintaining trust. We emphasise that ‘listening’ occurs only in the GMC guidelines.

The ‘Physician Charter’ [10], which describes a set of principles to which all medical professionals should adhere, had the lowest similarity to our results. While our doctor types cover all fundamental principles and professional responsibilities, several important aspects are missing from the Charter: themes such as listening, taking time, assertiveness, resilience, and online availability are absent from this set of medical principles.

Our results show general agreement regarding the essential characteristics of a good physician with both the public’s and the physicians’ competency frameworks. However, the public selected items that related to communication, personality, and social competence for high importance ratings, whereas these themes are almost absent from the existing frameworks. This disparity is significant, as it may reflect a shift in healthcare needs. Traditionally, physicians have played a paternalistic role, and the patients they care for have been passive recipients [24]. Today, the public expects more information and education than in the past. As members of the public become increasingly interested in playing an active and autonomous role in their healthcare decisions, physicians need to pay attention to their own ability to communicate effectively and empathetically. The importance ascribed by the public to their doctor having these skills suggests that more training in communication skills should be included in medical education.

Many concepts of professionalism include empathy when describing a good physician. Based on our results, the general population also desires friendliness, high social competence, and personality traits such as patience and optimism. Furthermore, good physicians should have ‘time for caring and listening’. Ensuring patient satisfaction has been shown to promote compliance and health-promoting behaviours, and it improves overall health outcomes [30]. As healthcare priorities shift towards communicative care, the human element of medicine may become more important than the technical aspects. At the same time, the strong public requirement for physicians to stay up to date underscores the importance of ongoing professional development [17].

The public also emphasised the importance of the availability and accessibility of doctors (e.g. offering house calls, opening hours on weekends, and in the evening). These items are implicit in the three frameworks, but are not formulated as explicitly as in our questionnaire. Statements relating to ‘digital doctoring’ (e.g. communication via e-mail; favouring digital medical files) were of medium importance to the respondents. As more and more people turn to the Internet for their healthcare advice, there was little surprise in finding they would like greater digital access to their physicians. But, as others have pointed out, ‘connectivity need not come at the expense of professionalism’ [31]. The perspective of the general population was that online communication and technology present increased opportunities for professionalism. They offer innovative ways of interacting and can have a positive impact on the relationship between physicians and the public. We recognize that the landscape of communication and collaboration will continue to change with technological and societal trends, and the ways in which both patients and physicians use websites will continue to evolve [32]. The existing guidelines served as a valuable starting point, but they need to be modified and adapted as technology advances and best practice continues to develop. Physicians need to become familiar with the relevant technologies to help both themselves and their patients navigate the online terrain.

This study has some powerful aspects. It included numerous respondents from different social backgrounds. Nevertheless, some limitations must be acknowledged. A possible limitation could be the use of a quota rather than a random sample. However, validity was achieved via representative sampling. We cannot eliminate the possibility that the sampling selection may have led to some bias, although we consider this contingency unlikely.

The sample was selected randomly and corresponded to the distribution of the Austrian population as a whole in terms of the sociodemographic characteristics age, gender, level of education, place of residence, and province. However, there might be a risk that unintentional selection has occurred. Thus, there is a possibility of underestimation or overestimation of correlations.

In addition, we hope that our research will stimulate future validation studies or other investigations of the described factors. Such a study should use confirmatory factor analysis and possibly can involve a Monte Carlo simulation to determine the probability distribution of the numbers of factors.

## Conclusion

In Austria, as in other European countries, there are increasing efforts to tailor medical care to the expectations of patients. In addition to the roles played by the medical profession and the health insurance companies, the general population can make a meaningful contribution to shaping the health system so as to provide the medical services they expect. However, information is necessary for this co-determination to work and there must be clarity about the multiple facets of attitudes to medicine. Since there is little such data for Austria, an attempt was made to obtain a multi-layered picture of the professional profile of a good doctor from the perspective of the general population.

The practice of medicine today faces unprecedented challenges. These centre increasingly on disparities between the people being cared for and the resources available to meet their needs. The rising demands of healthcare systems put pressure on physicians to abandon their traditional primary commitments to the patient’s interests [10]. Physicians need to reaffirm their commitment to the principles of professionalism, which should include a commitment to the well-being of individual patients together with an effort to collectively improve health benefits for society. Our identification of the different types strives to encourage commitment while promoting an agenda for the medical profession that is universal in scope and purpose.

All physicians should reflect constantly on their role in society: what both patients and society require, how care and attention should be interpreted through application, and how professionalism and interpersonal relationships can be reconciled. Physicians, medical practices, hospitals, and medical associations should try to create a system in which professionalism is lived out and experienced by all people coming into contact with the health services.

## Data Availability

The datasets used and/or analysed during the current study are available from the corresponding author on reasonable request.
